# Angiotensin II Reduces Cardiac AdipoR1 Expression through AT1 Receptor/ROS/ERK1/2/c-Myc Pathway

**DOI:** 10.1371/journal.pone.0049915

**Published:** 2013-01-22

**Authors:** Li Li, Zhi-Guo Zhang, Hong Lei, Cheng Wang, Li-Peng Wu, Jin-Yu Wang, Feng-Ying Fu, Wei-Guo Zhu, Li-Ling Wu

**Affiliations:** 1 Department of Physiology and Pathophysiology, Peking University Health Science Center, and Key Laboratory of Molecular Cardiovascular Sciences, Ministry of Education, Beijing, China; 2 Department of Biochemistry and Molecular Biology, Peking University Health Science Center, Beijing, China; 3 Key Laboratory of Cardiovascular Molecular Biology and Regulatory Peptides, Ministry of Health, Beijing, China; King's College London, University of London, United Kingdom

## Abstract

Adiponectin, an abundant adipose tissue-derived protein, exerts protective effect against cardiovascular disease. Adiponectin receptors (AdipoR1 and AdipoR2) mediate the beneficial effects of adiponectin on the cardiovascular system. However, the alteration of AdipoRs in cardiac remodeling is not fully elucidated. Here, we investigated the effect of angiotensin II (AngII) on cardiac AdipoRs expression and explored the possible molecular mechanism. AngII infusion into rats induced cardiac hypertrophy, reduced AdipoR1 but not AdipoR2 expression, and attenuated the phosphorylations of adenosine monophosphate-activated protein kinase and acetyl coenzyme A carboxylase, and those effects were all reversed by losartan, an AngII type 1 (AT1) receptor blocker. AngII reduced expression of AdipoR1 mRNA and protein in cultured neonatal rat cardiomyocytes, which was abolished by losartan, but not by PD123319, an AT2 receptor antagonist. The antioxidants including reactive oxygen species (ROS) scavenger NAC, NADPH oxidase inhibitor apocynin, Nox2 inhibitor peptide gp91 ds-tat, and mitochondrial electron transport chain complex I inhibitor rotenone attenuated AngII-induced production of ROS and phosphorylation of extracellular signal-regulated kinase (ERK) 1/2. AngII-reduced AdipoR1 expression was reversed by pretreatment with NAC, apocynin, gp91 ds-tat, rotenone, and an ERK1/2 inhibitor PD98059. Chromatin immunoprecipitation assay demonstrated that AngII provoked the recruitment of c-Myc onto the promoter region of AdipoR1, which was attenuated by PD98059. Moreover, AngII-induced DNA binding activity of c-Myc was inhibited by losartan, NAC, apocynin, gp91 ds-tat, rotenone, and PD98059. c-Myc small interfering RNA abolished the inhibitory effect of AngII on AdipoR1 expression. Our results suggest that AngII inhibits cardiac AdipoR1 expression *in vivo* and *in vitro* and AT1 receptor/ROS/ERK1/2/c-Myc pathway is required for the downregulation of AdipoR1 induced by AngII.

## Introduction

Adiponectin is an abundant adipose tissue-derived protein with important metabolic modulation and energy homeostasis effects [1]. Adiponectin participates in the regulation of cardiovascular function and its circulating level may be a predictor of cardiovascular outcomes [2]. For instance, high plasma adiponectin levels are associated with a reduced risk of myocardial infarction in men, whereas low plasma adiponectin levels are found in patients with coronary artery disease [3]. Plasma adiponectin concentration is significantly lower in hypertensive patients than that in normotensive men, which indicates that hypoadiponectinemia is an independent risk factor for hypertension [4]. There is growing evidence to demonstrate a negative correlation between circulating adiponectin and cardiac hypertrophy [5], [6]. Pressure overload in adiponectin-deficient mice results in enhanced concentric cardiac hypertrophy and adenovirus-mediated supplementation of adiponectin protects against the development of cardiac hypertrophy [7]. Therefore, adiponectin is an important endogenous adipokine protecting against cardiovascular disease.

Two types of adiponectin receptors (AdipoRs), AdipoR1 and AdipoR2, mediate most effects of adiponectin via activating adenosine monophosphate-activated protein kinase (AMPK) [8]. Downregulation of AdipoRs may play a role in metabolic syndrome and cardiovascular disease. Decreased expressions of AdipoR1 and AdipoR2 are found in skeletal muscle and adipose tissue of *ob-/ob-* mice [9] and in aortic tissues of rats fed with high-fat diet [10]. Expression of AdipoR1 is significantly decreased in infarcted mice heart [11]. AdipoRs also contribute to the inhibitory effect of adiponectin on endothelin-1- induced hypertrophy in cultured cardiomyocytes [12]. However, expression of AdipoRs in the process of cardiac remodeling has not been fully evaluated.

Angiotensin II (AngII), the major component of renin-angiotensin system (RAS), exerts vasoconstrictive, growth-promoting, and remodeling effects on the cardiovascular system [13]. Lower plasma adiponectin concentrations in patients with essential hypertension are elevated when administrated with AngII type 1 receptor (AT1) blocker or angiotensin converting enzyme inhibitor (ACEI) [14]. AngII infusion into rats decreases plasma concentration of adiponectin and adiponectin mRNA expression in adipose tissue [15]. These observations elicit that AngII is involved in the regulation of adiponectin synthesis and secretion. However, whether AngII interferes with cardiac adiponectin signaling cascade by regulating the expression of AdipoRs and its underlying mechanism is unknown.

The present study was designed to investigate the effect of AngII on AdipoRs expression in rats exposed to continuous infusion of AngII and in cultured neonatal rat cardiomyocytes. We also explored the possible molecular mechanism by which AngII regulates AdipoRs expression.

## Materials and Methods

### Materials

AngII, PD123319, CGP42112A, N-acetyl cysteine (NAC), apocynin, retenone, allopurinol, PD98059, SB202190, and SP600125 were purchased from Sigma-Aldrich Co. (St. Louis, MO, USA). Losartan was from Merck & Co. (Whitehouse Station, NJ, USA). gp91 ds-tat and scrambled gp91 ds-tat were from Anaspec (San Jose, CA, USA). Antibodies for AdipoR1, AdipoR2, phospho- and total extracellular signal-regulated kinase 1/2 (ERK1/2), nuclear factor (NF)-κB, and actin were from Santa Cruz Biotechnology (Santa Cruz, CA, USA). Antibodies for phospho- and total AMPK, and phospho- and total acetyl coenzyme A carboxylase (ACC) were from Cell Signaling Technology (Beverly, MA, USA). Anti-c-Myc antibody was from Upstate (Billerica, MA, USA). Anti-signal transducer and activator of transcription (STAT) 5 antibody was from Abcam (Cambridge, UK).

### In vivo rat model of continuous AngII infusion

All experimental procedures were approved by the Ethics Committee of Animal Research, Peking University Health Science Center, and the investigation conformed to the guidelines of the National Institutes of Health for the care and use of laboratory animals. Male Sprague-Dawley (SD) rats weighing 250 to 280 g were randomly divided into control, AngII, and losartan groups. An osmotic mini-pump (model 2004, Durect Corp., Cupertino, CA, USA) was subcutaneously embedded in rats under anesthesia with sodium pentobarbital (50 mg/kg, IP). AngII (400 ng/kg/min) or normal saline was infused constantly for 28 days. In the losartan group, rats also received losartan (10 mg/kg/day orally) during AngII infusion. At day 28, rats were anesthetized by sodium pentobarbital (50 mg/kg, IP). The right carotid artery was cannulated with polyethylene tubing connected to a Model TCB-500 transducer control unit (Millar Instruments, Houston, TX, USA). Hemodynamic parameters were recorded on a PowerLab data-acquisition system (ADInstruments, Sydney, Australia). Blood was collected and plasma was separated by centrifugation. Hearts, blood vessels, skeletal muscle, and adipose tissue were then excised for further investigation.

### Enzyme-linked immunosorbent assay (ELISA)

Plasma total adiponectin was determined with a rat adiponectin ELISA kit (Phoenix Pharmaceuticals Inc., Belmont, CA, USA).

### Culture of neonatal rat ventricular myocytes

Primary neonatal rat ventricular myocytes (NRVMs) was cultured as described previously [16]. Briefly, ventricles of 1–3 day-old SD rats were minced and digested in phosphate-buffered saline (PBS) containing 0.1% trypsin (Invitrogen Co., Carlsbad, CA, USA) and 0.05% type I collagenase (Invitrogen) at 37°C. Cells were pelleted and suspended in Dulbecco's modified Eagle's medium (DMEM) containing 10% fetal bovine serum (FBS, Invitrogen). A single preplating step was used to allow fibroblasts to attach to culture plates. Non-adherent cardiomyocytes were cultured for 3 to 4 days and placed in serum-free medium for 24 h before experiments. The identity of NRVMs was confirmed by morphological examination and by staining with anti-sarcomeric α-actin antibody; most (>95%) of the cells were identified as NRVMs.

### Quantitative real-time RT-PCR (qRT-PCR)

Total RNA of myocardial tissues or NRVMs was isolated by use of Trizol reagent (Invitrogen) and cDNA was generated from total RNA by use of the RevertAid First Strand cDNA Synthesis Kit (Fermentas, Burlington, ON, Canada). qRT-PCR was performed using following primer sets: AdipoR1 (forward, 5′-GGTTCTTCCTCATGGCTGTGA-3′, reverse, 5′-AGGTGAAGATACCCCACAGGT-3′), AdipoR2 (forward, 5′-GGCCTCCTCTTGGGATCTACT-3′, reverse, 5′-GTTGGCGCATGGTACTCATAC-3′) and β-actin (forward, 5′-ATGTTTGTGATGGGCGTGAACC-3′, reverse, 5′-CCCAGCATCGAAGGTAGAGGA-3′). Amplifications were performed in 35 cycles using an opticon continuous fluorescence detection system (MJ Research Inc., Waltham, MA, USA) with SYBR green fluorescence (Molecular Probes, Eugene, OR, USA). Each cycle consisted of a 45 s at 94°C, a 45 s at 56°C, and a 60 s at 72°C. All data were quantified by use of the comparative CT method, normalized to β-actin.

### Western blot analysis

Myocardial tissues or NRVMs were lysed in a buffer containing 50 mM Tris-HCl, pH 7.2, 0.1% sodium deoxycholate, 1% Triton X-100, 5 mM EDTA, 5 mM EGTA, 150 mM NaCl, 40 mM NaF, 2.175 mM sodium orthovanadate, 0.1% SDS, 0.1% aprotinin, and 1 mM phenylmethylsulfonyl fluoride. The lysates were centrifuged at 10 000×g for 10 min (4°C) and the supernatant was collected. Equal amounts of protein (50 µg), assayed using the Lowry's method [17], underwent SDS-PAGE and were transferred to polyvinylidene difluoride membranes. Membranes were incubated with primary antibodies and then probed with horseradish peroxidase-conjugated secondary antibodies. Blots were visualized using an enhanced chemiluminescence kit (Amersham Biosciences Inc., Piscataway, NJ, USA). The densities of bands were quantified using Quantity One software (Bio-Rad, Hercules, CA, USA).

### Immunohistochemical staining

NRVMs were fixed in 4% paraformaldehyde and permeabilized in 0.2% Triton X-100 in PBS. NRVMs were stained with anti-AdipoR1 or AdipoR2 antibody overnight at 4°C, then fluorescence-labeled secondary antibody for 2 h at 37°C. Nuclei were stained with 4, 6-diamidino-2-phenylindole (DAPI, Sigma-Aldrich). Fluorescence images were captured on the Olympus confocal microscope (Olympus Corp., Tokyo, Japan). AdipoRs-positive areas were quantified by use of Image J software (National Institute of Health, Bethesda, MD, USA). Fluorescence density of AdipoRs was expressed as the positive area corrected for the number of nuclei.

Ventricles were fixed in 10% phosphate-buffered formalin and embedded in paraffin. Deparaffined sections (5 µm thickness) were incubated with primary antibody against AdipoR1 or AdipoR2 overnight at 4°C, then horseradish peroxidase (HRP)-conjugated secondary antibody. Immunoreactions were visualized with 3-3′ diaminobenzidine tetrahydrochloride. The nuclei were counterstained with hematoxylin. Negative controls involved omission of primary antibodies. Microscopy images were analyzed by use of Leica QWin image analysis software (Leica, Wetzlar, Germany). Staining density was expressed as positive area corrected for the numbers of nuclei.

### Analysis of reactive oxygen species (ROS)

Intracellular ROS level was determined by using the cell-permeable, redox-sensitive fluorophore, 2′,7′-dichlorofluorescin diacetate (DCF-DA) (Molecular Probes, Eugene, OR, USA). NRVMs were incubated with 20 µM DCF-DA for 30 min and DCF fluorescence was visualized using the Olympus confocal microscope and analyzed with Image J software. All experiments were done with minimal exposure to light, and fluorescence was normalized to cell count.

### Chromatin immunoprecipitation (ChIP) assay

ChIP assay was performed as described by Wu *et al* [18]. Briefly, NRVMs were cross-linked with 1% formaldehyde, and collected into the lysis buffer (1% SDS, 10 mM EDTA, 50 mM Tris-HCl, pH 8.1, 1× protease inhibitor cocktail). After sonication, the lysates underwent immunoclearing and were immunoprecipitated with anti-c-Myc, anti-STAT5, or anti-NF-κB antibody, and equivalent concentration of normal rabbit IgG (Santa Cruz) served as negative control. After incubation with protein A Sepharose and salmon sperm DNA and a sequential washing, precipitates were heated at 65°C for 6 h to reverse the formaldehyde cross-linking. DNA fragments were purified with TIANquick Midi Purification Kit (Tiangen Biotech Co., Ltd., Beijing, China). PCR analysis was performed using the following primer sets: c-Myc site 1 (forward, 5′-TGTAGCTGTCTTCAGACACACCA-3′, reverse, 5′-TGTCTTTATTTGGGCTGGAGAG-3′), c-Myc site 2 (forward, 5′-ATGAGAGAGAAGGGCTGGAGA-3′, reverse, 5′-TCGCTGTCTTCAGACACACCA-3′), c-Myc site 3 (forward, 5′-AGATGGCTCAGCGGTTAAGAG-3′, reverse, 5′-TGTAGCTGTCTTCAGATACACCAGA-3′), STAT5 site 1 (forward, 5′-AGCCAAGCCTGTAACTCAGCA-3′, reverse, 5′-CAGGACCTCTGGAGGAGCAGT-3′), STAT5 site 2 (forward, 5′-TGAGAGAGAGAGAGAGCCAGGA-3′, reverse, 5′-TCAGGCTCTGCCAACTACTGAC-3′), and NF-κB (forward, 5′-AGCCAGCCAGGCCGTAAGTGT-3′, reverse, 5′-CCGGAAATGTTTACGGTGGAC-3′).

### Electrophoretic mobility shift assay (EMSA)

Nuclear protein fractions were separated as described [19]. Detection of DNA-protein interactions involved the nonisotopic method of the LightShift™ chemiluminescent EMSA kit (Pierce Biotechnology, Rockford, IL, USA). Briefly, nuclear extract protein was incubated with biotin-labeled DNA probes containing the consensus sequence 5′- AGAGATTCAACCACATGGTGCAGCTAG-3′ (sense). After reaction, DNA-protein complexes underwent 6% native PAGE and were transferred to a nitrocellulose membrane, then cross-linked to the membrane at 120 mJ/cm^2^ and detected by use of horseradish peroxidase-conjugated streptavidin according to the manufacturer's instructions.

### Small interfering RNA (siRNA)

NRVMs were seeded at a density of 3×10^5^ cells per well of six-well plates. After 48–72 h culture, cells were transfected with siRNA of interest by use of Lipofectamine 2000 (Invitrogen). The potent siRNA for rat c-Myc was designed by use of the siRNA Target Finder (Ambion Inc., Austin, TX, USA), based on the Rattus norvegicus mRNA sequences deposited at the NIH-PubMed database, and were submitted to BLAST analysis to assure specificity. The potent rat c-Myc siRNA 5′-GAAGAACAAGAUGAUGAGGTT-3′ (sense) and a nonspecific control siRNA were synthesized by GeneChem Co. (Shanghai, China).

### Hematoxylin and eosin staining

Deparaffined heart sections (5 µm thickness) were stained with hematoxylin and eosin for routine histological examination. Images were captured from six hearts in each group and cardiac myocyte cross-sectional surface area was evaluated by use of Leica QWin image analysis software. One hundred myocytes per heart were counted, and the average area was determined.

### Statistical analysis

Data are expressed as mean ± SE. Data were compared by one-way ANOVA for multiple groups, followed by Bonferroni's tests by use of GraphPad Prism 5.0 (GraphPad Software, San Diego, CA, USA). *P*<0.05 was considered statistically significant.

## Results

### 1. AngII infusion induces cardiac hypertrophy, reduces plasma adiponectin levels, inhibits cardiac AdipoR1 expression, and decreases phosphorylation of AMPK and ACC *in vivo*

#### 1.1 Effect of AngII on cardiac hypertrophy and ERK1/2 phosphorylation

Hemodynamic parameters on day 28 were shown in Table S1. Systolic blood pressure (SBP), diastolic blood pressure (DBP), d*P*/d*t* max, d*P*/d*t* min and left ventricular end-diastolic pressure (LVEDP) were increased in AngII-treated rats compared with those in controls (*P*<0.01), but were all suppressed by losartan, a specific AngII type 1 (AT1) receptor blocker. The cross-sectional surface area of cardiac myocytes (Figure S1A) and the ratio of heart weight to body weight (HW/BW, Figure S1B) were significantly increased (*P*<0.01) in AngII group. These results indicated that AngII infusion for 28 days resulted in cardiac hypertrophy. In addition, AngII infusion enhanced the phosphorylation of ERK1/2, a critical mediator in controlling the hypertrophic response (Figure S1C). Losartan attenuated AngII-induced cardiac hypertrophy and ERK1/2 phosphorylation (Figure S1A–C).

#### 1.2 Effect of AngII on plasma adiponectin levels, myocardial AdipoRs expression, and phosphorylations of AMPK and ACC

**Figure 1:**
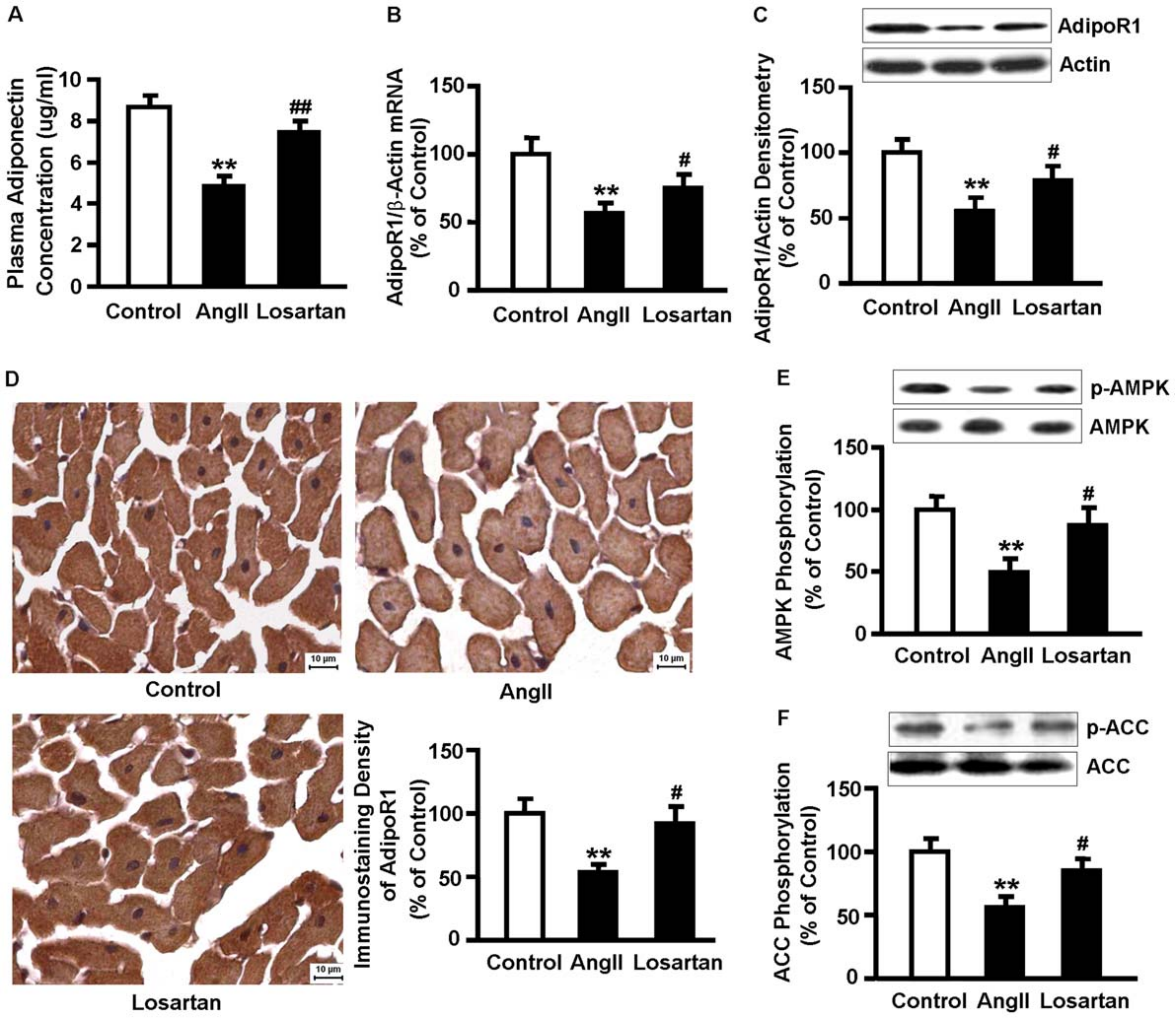
AngII infusion reduces plasma adiponectin levels, inhibits cardiac AdipoR1 expression, and decreases phosphorylation of AMPK and ACC. (A) Plasma adiponectin levels in the control, AngII, and losartan groups were detected by ELISA. (B) Levels of AdipoR1 mRNA were analyzed by qRT-PCR, and β-actin was used as an internal control. (C) Myocardial extracts were immunoblotted with antibody specific for AdipoR1. Blots were reprobed with actin to confirm equal loading. (D) Representative immunostaining images and averaged bar graphs of AdipoR1 density. AdipoR1 is shown in brown and cell nuclei in blue. Scale bar represents 10 µm (6 fields in each sample were scanned and averaged). (E) Myocardial extracts were immunoblotted with antibody specific for p-AMPKα at Thr-172. Blots were reprobed with antibody for AMPK to confirm equal loading. (F) Myocardial extracts were immunoblotted with antibody specific for p-ACC at Ser-79. Blots were reprobed with antibody for ACC to confirm equal loading. Data represent mean ± SE. n = 6 in each group. **P<0.01 vs. control. ^#^ *P*<0.05, ^##^ *P*<0.01 vs. AngII.

Plasma adiponectin levels in AngII-infused rats were significantly lower than that in controls, and losartan treatment prevented the AngII-induced decrease in circulating adiponectin levels (Figure 1A). Expression of AdipoR1 mRNA and protein in the AngII-induced hypertrophic heart was significantly decreased, which was reversed by losartan (Figure 1B and C). Immunostaining results showed that AdipoR1 was distributed widely in ventricular myocytes in control group. AngII infusion reduced the positive staining density of myocardial AdipoR1, which was reversed by losartan (Figure 1D). The negative control image was shown in Figure S2. Phosphorylations of AMPK-α at Thr-172 (Figure 1E) and ACC at Ser-79 (Figure 1F), a direct downstream target of AMPK, were attenuated in the hypertrophic hearts, and those effects were reversed by losartan administration. However, there was no significant difference of myocardial AdipoR2 mRNA and protein expression among the control, AngII, and losartan groups (Figure S3).

#### 1.3 Effect of AngII on AdipoRs expression in skeletal muscle, adipose tissue, and blood vessels

There was no significant difference of AdipoRs protein expression in skeletal muscle and adipose tissue among the control, AngII, and losartan groups (Figure S4A–D). AdipoR1 protein expression in blood vessels was increased by 45.2% in AngII group when compared with that in control group, which was attenuated by losartan treatment (*P*<0.05, Figure S4E). Expression of AdipoR2 protein in blood vessels did not change in AngII-infused rats (Figure S4F).

### 2. AngII decreases AdipoR1 expression in NRVMs

**Figure 2:**
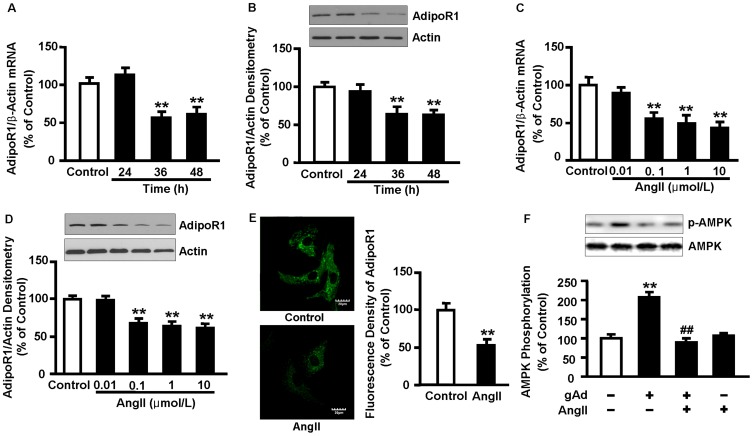
AngII decreases AdipoR1 expression in NRVMs. NRVMs were incubated with 0.1 μmol/L AngII for the indicated times, or treated with AngII at the indicated concentrations for 48 h. (A, C) Levels of AdipoR1 mRNA were analyzed by qRT-PCR. (B, D) Expression of AdipoR1 protein was determined by Western blot analysis. (E) Representative immunofluorescence images and averaged bar graphs of AdipoR1 density in unstimulated control NRVMs and cells treated with 0.1 µmol/L AngII for 48 h. Green fluorescence signals represent AdipoR1 protein. Scale bar represents 20 µm (6 fields in each sample were scanned and averaged). (F) NRVMs were pretreated with 0.1 µmol/L AngII for 48 h and then stimulated with 1 µg/ml globular adiponectin for 10 min. AMPK phosphorylation in cell lysates was determined by Western blot analysis. Data represent mean ± SE of three independent experiments. **P<0.01 vs. control. ^##^ *P*<0.01 vs. AngII.

To determine the effect of AngII on AdipoRs expression *in vitro*, NRVMs were incubated with 0.1 µmol/L AngII for the indicated times. Expression of AdipoR1 mRNA and protein was significantly decreased after exposure to AngII for 36 and 48 h (*P*<0.01, Figure 2A and B). Incubation with 0.1, 1, and 10 µmol/L AngII for 48 h decreased the level of AdipoR1 mRNA by 45.6% (*P*<0.01), 50.9% (*P*<0.01), and 56.9% (*P*<0.01), respectively (Figure 2C). AngII-reduced AdipoR1 protein expression was parallel with a decrease in its mRNA level (*P*<0.01, Figure 2D). Immunofluorescence staining showed that AdipoR1 was widespread in the cytoplasm and membrane in unstimulated control cells. The fluorescence densities were attenuated significantly after AngII incubation for 48 h (*P*<0.01, Figure 2E) AMPK phosphorylation was enhanced upon incubation with 1 µg/ml globular adiponectin for 10 min, and this effect was dramatically attenuated in AngII-pretreated NRVMs (Figure 2F). Expression of AdipoR2 had no change upon incubation with AngII (Figure S5).

### 3. The AngII-induced decrease in AdipoR1 expression is AT1 receptor-dependent

**Figure 3:**
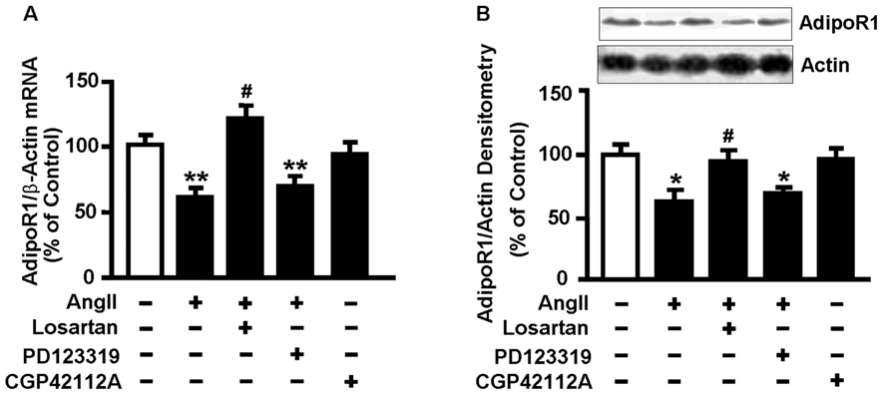
The AngII-induced decrease in AdipoR1 expression is AT1 receptor-dependent. NRVMs were pretreated with losartan (10 µmol/L) or PD123319 (1 µmol/L) for 1 h and then stimulated with 0.1 µmol/L AngII for 48 h, or incubated with CGP42112A (1 µmol/L) for 48 h. (A) Levels of AdipoR1 mRNA were analyzed by qRT-PCR, and β-actin was used as an internal control. (B) Western blot was performed to detect levels of AdipoR1 and actin protein expression. Data represent mean ± SE of three independent experiments. *P<0.05, **P<0.01 vs. control. ^#^ *P*<0.05 vs. AngII.

To determine the receptor subtypes mediating the inhibitory effect of AngII on AdipoR1 expression, NRVMs were pretreated with 10 µmol/L losartan or 1 µmol/L PD123319, an AT2 receptor antagonist, for 1 h and then stimulated with 0.1 µmol/L AngII for 48 h. Expression of AdipoR1 mRNA and protein was downregulated by AngII, which was reversed significantly when pretreated with losartan. However, the suppressive effect of AngII on AdipoR1 expression was not affected by PD123319. Furthermore, CGP42112A, a specific AT2 receptor agonist, did not change the expression of AdipoR1 (Figure 3A and B). Losartan and PD123319 alone had no effect on AdipoR1 expression (data not shown).

### 4. The AngII-induced decrease in AdipoR1 expression involves ERK1/2 activation and ROS production

#### 4.1 Effect of mitogen-activated protein kinase inhibitors on AngII-reduced AdipoR1 expression

**Figure 4:**
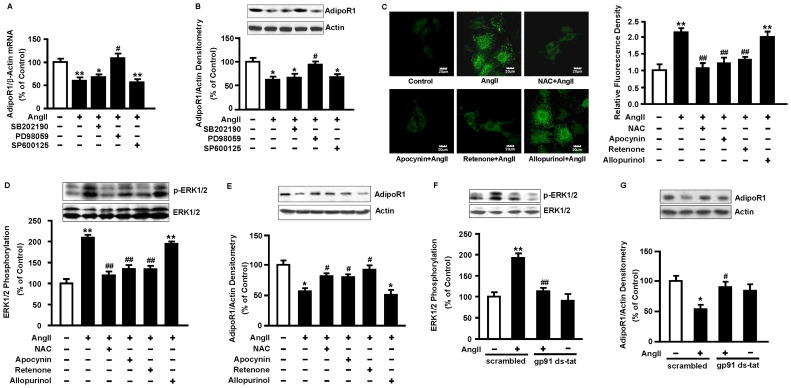
The AngII-induced decrease in AdipoR1 expression involves ERK1/2 activation and ROS production. (A, B) NRVMs were pretreated with SB202190 (5 µmol/L), PD98059 (10 µmol/L), or SP600125 (10 µmol/L) for 1 h, then stimulated with 0.1 µmol/L AngII for 48 h. Expression of AdipoR1 mRNA and protein was analyzed by qRT-PCR and Western blot respectively. (C) Representative fluorescence images and histogram of relative fluorescence density of ROS. NRVMs were pretreated with NAC (10 mmol/L), apocynin (100 µmol/L), rotenone (10 µmol/L), or allopurinol (100 µmol/L) for 1 h, followed by DCF-DA incubation for 30 min, and then stimulated with 0.1 µmol/L AngII for 30 min. DCF fluorescence was visualized using confocal microscopy. The green color represents ROS and the fluorescence density was normalized to cell count. Scale bar represents 20 µm (6 fields in each sample were scanned and averaged). (D, E) NRVMs were pretreated with NAC (10 mmol/L), apocynin (100 µmol/L), rotenone (10 µmol/L), or allopurinol (100 µmol/L) for 1 h and then stimulated with 0.1 µmol/L AngII for 30 min (D) or 48 h (E). Western blot was performed to detect levels of p-ERK1/2 (D) or AdipoR1 (E). Blots were reprobed with antibody for ERK1/2 (D) or actin (E) to confirm equal loading. (F, G) NRVMs were pretreated with 10 µmol/L gp91 ds-tat or scrambled gp91 ds-tat for 1 h and then stimulated with 0.1 µmol/L AngII for 30 min (F) or 48 h (G). Western blot was performed to detect levels of p-ERK1/2 (F) or AdipoR1 (G). Data represent mean ± SE of three independent experiments. *P<0.05, **P<0.01 vs. control. ^#^ *P*<0.05, ^##^ *P*<0.01 vs. AngII.

To explore the role of mitogen-activated protein kinase (MAPK) in AngII-reduced AdipoR1 expression, we employed SB202190 (5 µmol/L), a selective p38MAPK inhibitor, PD98059 (10 µmol/L), an ERK1/2 upstream kinase inhibitor, and SP600125 (10 µmol/L), a c-Jun-N-terminal kinase (JNK) inhibitor to preincubate NRVMs for 1 h, and then stimulated with 0.1 µmol/L AngII for 48 h. PD98059 reversed AngII-reduced AdipoR1 expression. SB202190 and SP600125 did not affect AngII-induced AdipoR1 downregulation (Figure 4A and B). Each of the inhibitors alone had no effect on AdipoR1 expression (Figure S6A).

#### 4.2 Effect of the pharmacological inhibition of ROS on AngII-induced ERK1/2 phosphorylation and AdipoR1 downregulation

We explored whether ROS was involved in the activation of ERK1/2 and downregulation of AdipoR1 in response to AngII. Fluorescence density of DCF was significantly increased after AngII treatment for 30 min. AngII-increased ROS production was eliminated by the ROS scavenger NAC (10 mmol/L), the NADPH oxidase inhibitor apocynin (100 µmol/L), and the inhibitor of the mitochondrial electron transport chain complex I rotenone (10 µmol/L), but not by the inhibitor of xanthine oxidase allopurinol (100 µmol/L) (Figure 4C). AngII-mediated ERK1/2 phosphorylation was also attenuated by the antioxidants including NAC, apocynin, and rotenone, but not allopurinol (Figure 4D). Similarly, the inhibitory effect of AngII on AdipoR1 expression was reversed by NAC, apocynin, and rotenone, but not by allopurinol (Figure 4E). Each of the inhibitors alone had no effect on ERK1/2 phosphorylation and AdipoR1 expression (Figure S6B and C).

#### 4.3 Effect of Nox2 inhibitor on AngII-induced ERK1/2 phosphorylation and AdipoR1 downregulation

The catalytic subunit gp91phox (Nox2), a major isoform of NADPH oxidase expressed in cardiomyocytes, is critical for cardiac hypertrophy in response to chronic AngII infusion [20]. We then employed gp91 ds-tat, a Nox2 inhibitor peptide, to examine the role of Nox2 in this process. Pretreatment with gp91 ds-tat attenuated AngII-induced ROS production (Figure S7) and was associated with an 81.2% reduction in ERK1/2 phosphorylation induced by AngII (*P*<0.01, Figure 4F). AngII-reduced AdipoR1 expression was reversed markedly by gp91 ds-tat (*P*<0.05, Figure 4G).

### 5. AngII promotes binding of c-Myc to the AdipoR1 promoter region and it requires ERK1/2 involvement

**Figure 5:**
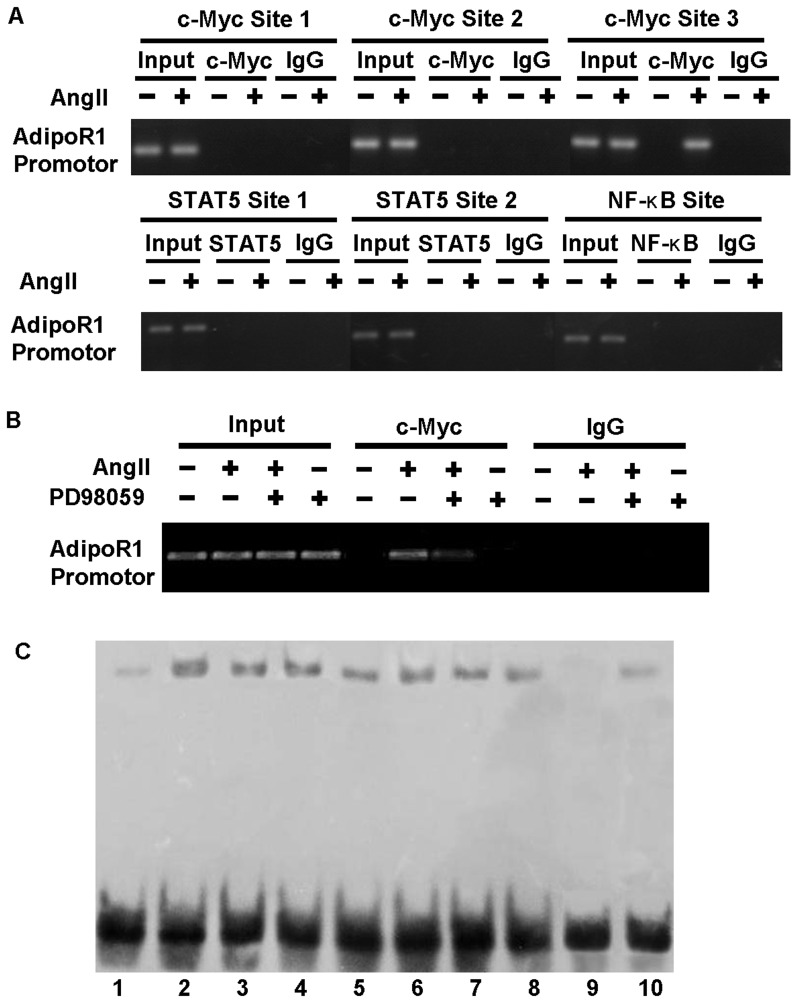
AngII promotes binding of c-Myc to the AdipoR1 promoter region and it requires ERK1/2 involvement. (A) NRVMs were stimulated with 0.1 µmol/L AngII for 48 h. Sheared chromatin prepared from cardiomyocytes was immunoprecipitated with the indicated antibodies (control IgG, anti-c-Myc, anti-STAT5 or anti-NF-κB antibodies). The immunoprecipitated DNA was used as a template for PCR using the indicated primer sets. (B) NRVMs were pretreated with 10 µmol/L PD98059 for 1 h, then stimulated with 0.1 µmol/L AngII for 48 h. Chromatin fragments were immunoprecipitated with anti-c-Myc antibody or normal rabbit IgG. Immunoprecipitated chromatin was quantified with RT-PCR. (C) NRVMs were pretreated with losartan (10 µmol/L, lane 3), NAC (10 mmol/L, lane 4), apocynin (100 µmol/L, lane 5), rotenone (10 µmol/L, lane 6), gp91 ds-tat (10 µmol/L, lane 7) or PD98059 (10 µmol/L, lane 8) for 1 h, and then stimulated with 0.1 µmol/L AngII for 48 h. DNA binding activity of c-Myc was determined by EMSA with nuclear extracts of NRVMs and DNA probes. Lane 1, control; lane 2, AngII; lane 3–8, inhibitors; lane 9, with 100-fold molar excess of unlabeled oligonucleotide; lane 10, with 100-fold molar excess of unlabeled mutant oligonucleotide.

To further reveal the transcription mechanism of AngII-mediated AdipoR1 expression, we screened the DNA sequence of rat AdipoR1 promoter region by use of Genomatix from the UCSC Genome Browser Database (http://www.genome.ucsc.edu) and identified six putative binding sites located between −3000 and −200 on AdipoR1 promoter sequence for three cardiovascular-related transcription factors, including three for c-Myc, two for STAT5, and one for NF-κB (Table 1). ChIP assay showed that AngII provoked the recruitment of c-Myc to its putative binding site 3 between −2515 and −2503 located on the AdipoR1 promoter region. AngII did not affect the binding of c-Myc to sites 1 and 2, and had no influence on the binding of STAT5 and NF-κB to their corresponding putative binding sites (Figure 5A). PD98059 preincubation partially, but significantly, inhibited the binding of c-Myc to AdipoR1 promoter region induced by AngII (Figure 5B). EMSA results showed that AngII treatment for 48 h increased the DNA binding activity of c-Myc (lane 2). Enhanced c-Myc binding activity was significantly attenuated by losartan, NAC, apocynin, rotenone, gp91 ds-tat, and PD98059 (lane 3–8). Unlabeled wild-type oligonucleotide completely competed the control band off (lane 9), whereas unlabeled mutant oligonucleotide failed to compete (lane 10), demonstrating the specificity of the DNA-protein complex (Figure 5C).

**Table 1:** Putative binding sites of c-Myc, STAT5, and NF-κB in the promoter region of AdipoR1.

	Sequence	Chromosomal Location
c-Myc site 1	CaaccaCATGgtg	−1051 to −1039
c-Myc site 2	CaaccaCATGgtg	−2036 to −2024
c-Myc site 3	CaaccaCATGgtg	−2515 to −2503
STAT5 site 1	atccTTCTgcgaactgcac	−2636 to −2618
STAT5 site 2	aaaaTTCTtcgaatggtac	−2830 to −2812
NF-κB site	gcGGGActggccc	−225 to −213

### 6. AngII decreases AdipoR1 expression in a c-Myc-dependent manner

**Figure 6:**
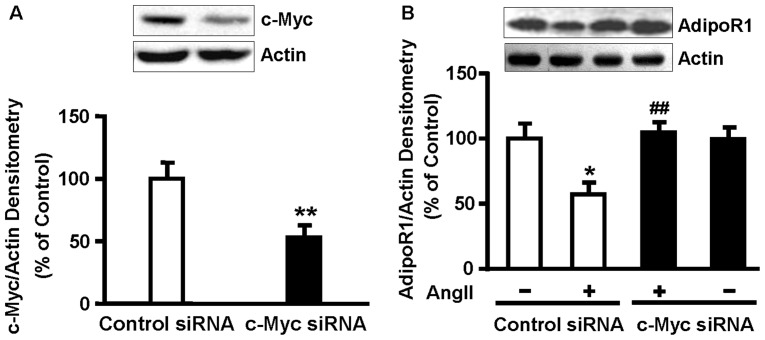
AngII decreases AdipoR1 expression in a c-Myc-dependent manner. (A) NRVMs were transfected with c-Myc or control siRNA (100 nmol/L) for 48 h. Western blot was performed to detect levels of c-Myc and actin. (B) NRVMs were transfected with c-Myc or control siRNA (100 nmol/L) for 48 h and serum-deprived for 24 h, followed by incubation with 0.1 µmol/L AngII for 48 h. The level of AdipoR1 protein was analyzed by Western blot analysis with actin as an internal control. Data represent mean ± SE of three independent experiments. *P<0.05, **P<0.01 vs. control. ^##^ *P*<0.01 vs. AngII.

To further examine the effect of c-Myc on AngII-reduced AdipoR1 expression, NRVMs were transfected with control or c-Myc siRNA (100 nmol/L) for 48 h and serum-deprived for 24 h, then incubated with 0.1 µmol/L AngII for 48 h. c-Myc siRNA greatly reduced c-Myc protein level (*P*<0.01, Figure 6A) and abolished the suppressive effect of AngII on AdipoR1 expression (*P*<0.01, Figure 6B). Transfection with c-Myc siRNA alone had no effect on protein expression of AdipoR1.

## Discussion

In the present study, we demonstrated that AngII reduced AdipoR1 but not AdipoR2 expression both in hearts of AngII-infused rats and in cultured neonatal rat cardiomyocytes. We also found that AT1 receptor, ROS derived from NADPH oxidase and mitochondria, and ERK1/2 were critical for AngII-reduced AdipoR1 expression. The recruitment of c-Myc to AdipoR1 promoter region was required for the transcription modulation of AngII on AdipoR1 expression. Moreover, the AT1 receptor/ROS/ERK1/2 pathway was involved in AngII-induced DNA binding activity of c-Myc. These results suggest that the downregulation of cardiac AdipoR1 by AngII may play an important role in the progression of cardiac remodeling. This research focused on the mechanism involved in AngII-reduced AdipoR1 expression in cardiomyocytes will hopefully lead to novel strategies for clinical cardioprotection.

Adiponectin is an endogenous protective protein that acts as a modulator in energy metabolism and cardiovascular function [1], [2]. Adiponectin and AdipoRs play important roles in alleviating pathological cardiac hypertrophy. Low levels of adiponectin are associated with a further progression of left ventricular hypertrophy in patients presenting with hypertension, left ventricular diastolic dysfunction and hypertrophy [21], [22]. Adiponectin-deficient mice have enhanced concentric cardiac hypertrophy and increased mortality in response to AngII infusion and aortic constriction, which is attenuated by adiponectin supplementation [7]. As the critical components in the adiponectin signaling cascade, AdipoRs are proved to mediate the protective action of adiponectin against insulin resistance, type 2 diabetes, and cardiomyocytes hypertrophy [12], [23]. A series of studies have indicate that AdipoR1 expression is downregulated in various organs under pathological conditions, such as in the pancreas and liver of *ob-/ob-* and *db-/db-* mice [24], in the adipose tissue of obese diabetic KKAy mice and patients with obesity [25], [26], in the soleus muscle of obese Zucker rats [27], and in hyperglycemia- and hyperinsulinemia- stimulated L6 myoblasts [28]. In type 2 diabetic rats induced by high-fat and high-sugar diets, cardiac hypertrophy and decreased heart function are associated with decreased myocardial AdipoR1 expression and AMPK phosphorylation [29]. Expression of AdipoR1 is also reduced in infarcted mice heart and tumor necrosis factor-α-treated cardiomyocytes [11]. Here, we provided evidence that AdipoR1 expression was decreased in hearts, increased in blood vessels, and did not change in skeletal muscle and adipose tissue in AngII-infused rats. Several studies have also shown the diverse action of hormones or drugs on AdipoRs expression in different tissues. Rosiglitazone treatment for 8 weeks improves the histological lesions in liver of nonalcoholic steatohepatitis rats, increases mRNA and protein expressions of AdipoR1 and AdipoR2 in liver and visceral fat, with down-regulation of the two receptors in muscle [30]. Leptin decreases AdipoR2 expression in rat liver and stomach, but increases its expression in adipose tissue [31]. The downregulation of AdipoR1 by AngII in cultured neonatal cardiomyocytes revealed the direct inhibitory effect of AngII on AdipoR1 expression. AdipoR2 expression did not change in our *in vivo* rat model and in cultured cardiomyocytes. Decreased AMPK phosphorylation indicated that the function of AdipoR1 was suppressed in hearts of AngII-infused rats and in AngII-treated cardiomyocytes. Previous studies have demonstrated that transfection of siRNA specific for AdipoR1 or AdipoR2 reverses the stimulatory effect of adiponectin on AMPK activation and the suppressive effect of adiponectin on endothelin-1-induced cellular hypertrophy in cardiomyocytes [12]. Considered together, our results suggest that the reduced AdipoR1 expression by AngII may contribute to the attenuated adiponectin signaling, thereby reducing the protective effect of adiponectin and resulting in abnormal metabolic change and cardiac hypertrophy.

AngII binds to and activates AT1 and AT2 receptors, which seem to induce phenotypically opposite responses [32]. It would be of interest to determine the exact subtype involved in the AngII-reduced AdipoR1 expression. The decreased expression of AdipoR1 by AngII was abolished by losartan, but not affected by PD123319. Furthermore, activation of AT2 receptor with CGP42112A had no influence on AdipoR1 expression. These results indicate that the inhibitory effect of AngII on AdipoR1 expression is mediated thoroughly by AT1 receptor. The prevention effect of losartan on the AngII-reduced AdipoR1 expression will provide further understanding of the pathogenesis of cardiac hypertrophy and provide novel insights into this anti-remodeling therapy.

AngII regulates cell behavior through several mechanisms downstream of AT1 receptor, including stimulation of MAPK cascades. ERK1/2, p38MAPK, and JNK are three major members of the MAPK family, but the exact signal molecule responsible for AdipoR1 expression in this process is still poorly understood. We found that the suppressive effect of AngII on AdipoR1 expression was prevented by PD98059, but not by SB202190 and SP600125, which indicates that ERK1/2 is critical for AngII-reduced AdipoR1 expression in cardiomyocytes.

Increased ROS production mediates the hypertrophic response in cardiomyocytes to stretch or to neurohormonal stimuli such as AngII [33]. Suppression of ROS formation inhibits AngII-induced cardiomyocyte hypertrophy [34]. There are four enzymatic systems including NADPH oxidase, xanthine oxidase, uncoupled NO synthase, and the mitochondrial electron transport chain that produce ROS predominantly in mammalian cells [35]–[37]. We showed here that AngII-stimulated ROS production in cardiomyocytes resulted mainly from NADPH oxidase and mitochondrial electron transport enzyme. The crucial role of NADPH oxidase and mitochondrial oxidative stress in AngII-induced cardiac hypertrophy has been implicated in a series of studies [33]. Recent evidence also indicates a linking between these two sources for ROS production. In cardiomyocytes and vascular smooth muscle cells, AngII enhances the production of ROS through the activation of NADPH oxidase, which in turn triggers mitochondrial ATP dependent K^+^ channel opening and mitochondrial ROS production [38], [39]. ROS has been proposed to result in ERK1/2 activation and modulate the subsequent development of cardiac hypertrophy in response to different stimuli such as pressure overload, serotonin, AngII, and isoproterenol [40]. We then explored the ROS-mediated signal transduction involved in AngII-reduced AdipoR1 expression. We found that NAC, apocynin, and rotenone blocked the increase of ERK1/2 phosphorylation and the decrease of AdipoR1 expression by AngII, but inhibition of xanthine oxidase with allopurinol did not affect AngII-induced ERK1/2 activation and AdipoR1 downregulation. Nox2 NADPH oxidase is normally quiescent in cardiomyocytes and is activated through binding the cytosolic regulatory components to generate superoxide anion by stimuli such as G protein-coupled receptor agonists, growth factors, and cytokines [41]. Nox2 plays an important role in the hypertrophic response to AngII and myocardial infarction [20], [42], [43]. Nox2 knock-out mice subjected to AngII infusion or myocardial infarction show a less extent of cardiac hypertrophy than the matched wild-type mice. The current study demonstrated that Nox2 inhibition with gp91 ds-tat attenuated AngII-induced ERK1/2 phosphorylation and reversed AngII-reduced AdipoR1 expression. These results confirm that NADPH oxidase, especially Nox2, and mitochondria are major sources for ROS production in response to AngII in cardiomyocytes and are critical for the subsequent ERK1/2 activation, thereby being required for AngII-reduced AdipoR1 expression.

Although AdipoRs expression is mediated by various physiological and pathological factors, the precise mechanism involved in AdipoRs transcription regulation has not been fully evaluated. Recently, an insulin-responsive repressor element, termed nuclear inhibitory protein, has been identified in the AdipoR1 promoter region of C2C12 cells [44]. Our previous study has demonstrated that insulin decreases cardiac AdipoR1 expression via the PI3K/Akt pathway and that FoxO1 plays an important role in insulin-mediated AdipoR1 transcription by binding directly to the AdipoR1 promoter [45]. In the present study, we identified the putative binding sites located on AdipoR1 promoter region for c-Myc, STAT5 and NF-κB which are activated by AngII [46], [47]. ChIP analysis provided the evidence that AngII provoked the binding of c-Myc, but not STAT5 or NF-κB, to the AdipoR1 promoter region in cardiomyocytes. The suppressive effect of AngII on AdipoR1 expression was abolished by c-Myc siRNA, suggesting that c-Myc acts negatively on AdipoR1 transcription. There is a growing body of evidence to implicate the crucial role of c-Myc in provoking cardiac hypertrophy *in vivo* and in cultured cardiomyocytes [48], [49]. Inactivation of c-Myc in the adult myocardium attenuates hypertrophic growth and decreases the expression of glycolytic and mitochondrial biogenesis genes in response to hemodynamic load, which implies that c-Myc is an important regulator of energy metabolism in the heart in response to hypertrophic stimulus [50]. Our data give the information that c-Myc plays an important role in the transcription regulation of AdipoR1 by AngII and provide an alternative mechanism about c-Myc activation in regulating hypertrophic response. A series of studies have indicated that ERK1/2 responds to different stimuli and mediates the expression and activation of c-Myc in a plethora of cell types; examples are AngII-induced c-Myc expression in astrocytes [51], and serum- or thrombin-induced c-Myc phosphorylation in vascular smooth muscle cells [52]. We demonstrated here that AT1 receptor, ROS generation, and ERK1/2 activation were required for AngII-induced DNA binding activity of c-Myc.

## Conclusions

In summary, we demonstrate that AngII reduces the expression of AdipoR1 but not AdipoR2 in hearts of AngII-infused rat model and in cultured neonatal cardiomyocytes. AngII decreases AdipoR1 expression through its AT1 receptor. ROS generated from NADPH oxidase, especially Nox2, and mitochondria and the sequential activation of ERK1/2 are involved in AngII-reduced AdipoR1 expression. Moreover, c-Myc plays an important role in AngII-mediated AdipoR1 transcription regulation in cardiomyocytes through binding to AdipoR1 promoter directly. The AT1 receptor/ROS/ERK1/2 pathway is required for AngII-induced DNA binding activity of c-Myc. These findings may improve our understanding of the molecular mechanisms involved in cardiac hypertrophy and provide new insights into future therapeutic targets for cardiac remodeling.

## Supporting Information

Figure S1 **AngII infusion induces cardiac hypertrophy and enhances ERK1/2 phosphorylation.** (A) Representative HE staining images and histogram of cardiac myocytes cross-sectional surface area in the control, AngII, and losartan groups (100 myocytes in each section were scanned and averaged). (B) Averaged bar graphs of HW/BW in the control, AngII, and losartan groups. (C) Myocardial extracts were immunoblotted with antibody specific for p-ERK1/2. Membranes were stripped and re-probed to normalize the blotted samples with anti-ERK1/2 antibody. Relative densities of p-ERK1/2 were quantified by scanning densitometry and normalized to percentage of control. Data represent mean ± SE. n = 6 in each group. **P<0.01 vs. control. ^##^ *P*<0.01 vs. AngII.
(TIF)

Figure S2 **The negative control image of AdipoR1 immunostaining.** Deparaffined heart sections (5 µm thickness) were incubated with PBS overnight at 4°C, then horseradish peroxidase (HRP)-conjugated secondary antibody. Immunoreactions were visualized with 3-3′ diaminobenzidine tetrahydrochloride. The nuclei were counterstained with hematoxylin. Cell nuclei is shown in blue. Scale bar represents 10 µm.
(TIF)

Figure S3 **AdipoR2 expression in the control, AngII, and losartan groups.** (A) Levels of AdipoR2 mRNA were analyzed by qRT-PCR, and β-actin was used as an internal control. (B) Expression of AdipoR2 protein was determined by Western blot analysis. (C) Representative immunostaining images and averaged bar graphs of AdipoR2 density. AdipoR2 is shown in brown and cell nuclei in blue. Scale bar represents 10 µm (6 fields in each sample were scanned and averaged). Data represent mean ± SE. n = 6 in each group.
(TIF)

Figure S4 **Effect of AngII on AdipoRs expression in skeletal muscle, adipose tissue, and blood vessels.** Protein lysates from skeletal muscle (A, B), adipose tissue (C, D), and blood vessels (E, F) were immunoblotted with antibody specific for AdipoR1 (A, C, E) or AdipoR2 (B, D, F). Blots were reprobed with actin to confirm equal loading. Data represent mean ± SE. n = 6 in each group. *P<0.05 vs. control. ^#^ *P*<0.05 vs. AngII.
(TIF)

Figure S5 **Effect of AngII on AdipoR2 expression in NRVMs.** (A, B) NRVMs were incubated with 0.1 µmol/L AngII for the indicated times, or treated with AngII at the indicated concentrations for 48 h. Levels of AdipoR2 mRNA were analyzed by qRT-PCR. (C) Representative immunofluorescence images and averaged bar graphs of AdipoR2 density in unstimulated control NRVMs and cells treated with 0.1 µmol/L AngII for 48 h. Green fluorescence signals represent AdipoR2 protein. Scale bar represents 20 µm (6 fields in each sample were scanned and averaged). Data represent mean ± SE of three independent experiments.
(TIF)

Figure S6 **Effect of pharmacological inhibitors of MAPK, ROS scavengers, and the specific inhibitors of ROS-producing enzymes on ERK1/2 phosphorylation or AdipoR1 expression.** (A) NRVMs were treated with SB202190 (5 µmol/L), PD98059 (10 µmol/L), or SP600125 (10 µmol/L) for 1 h. Western blot was performed to detect levels of AdipoR1 and actin. (B, C) NRVMs were treated with NAC (10 mmol/L), apocynin (100 µmol/L), rotenone (10 µmol/L), or allopurinol (100 µmol/L) for 1 h. Western blot was performed to detect levels of p-ERK1/2 (B) and AdipoR1 (C). Data represent mean ± SE of three independent experiments.
(TIF)

Figure S7 **Representative fluorescence images and histogram of relative fluorescence density of ROS.** NRVMs were pretreated with 10 µmol/L gp91 ds-tat or scrambled gp91 ds-tat for 1 h, followed by DCF-DA incubation for 30 min, and then stimulated with 0.1 µmol/L AngII for 30 min. DCF fluorescence was visualized using confocal microscopy. The green color represents ROS and the fluorescence density was normalized to cell count. Scale bar represents 20 µm (6 fields in each sample were scanned and averaged). Data represent mean ± SE of three independent experiments. **P<0.01 vs. control. ^##^ *P*<0.01 vs. AngII.
(TIF)

Table S1 **Hemodynamic parameters with control, angiotensin II, and losartan treatment.** AngII, angiotensin II; SBP, systolic blood pressure; DBP, diastolic blood pressure; d*P*/d*t* max, peak rate of left ventricular pressure increase; d*P*/d*t* min, peak rate of left ventricular pressure decrease; LVEDP, left ventricular end-diastolic pressure. Data are mean ± SE. **P<0.01 vs. control. ^##^ *P*<0.01 vs. AngII.
(DOC)
