# Supplementary figures and images for: Angiotensin II Reduces Cardiac AdipoR1 Expression through AT1 Receptor/ROS/ERK1/2/c-Myc Pathway
Source: PLoS One. 2013 Jan 22;8(1):e49915. doi: 10.1371/journal.pone.0049915 (PMC3551944; doi:10.1371/journal.pone.0049915)

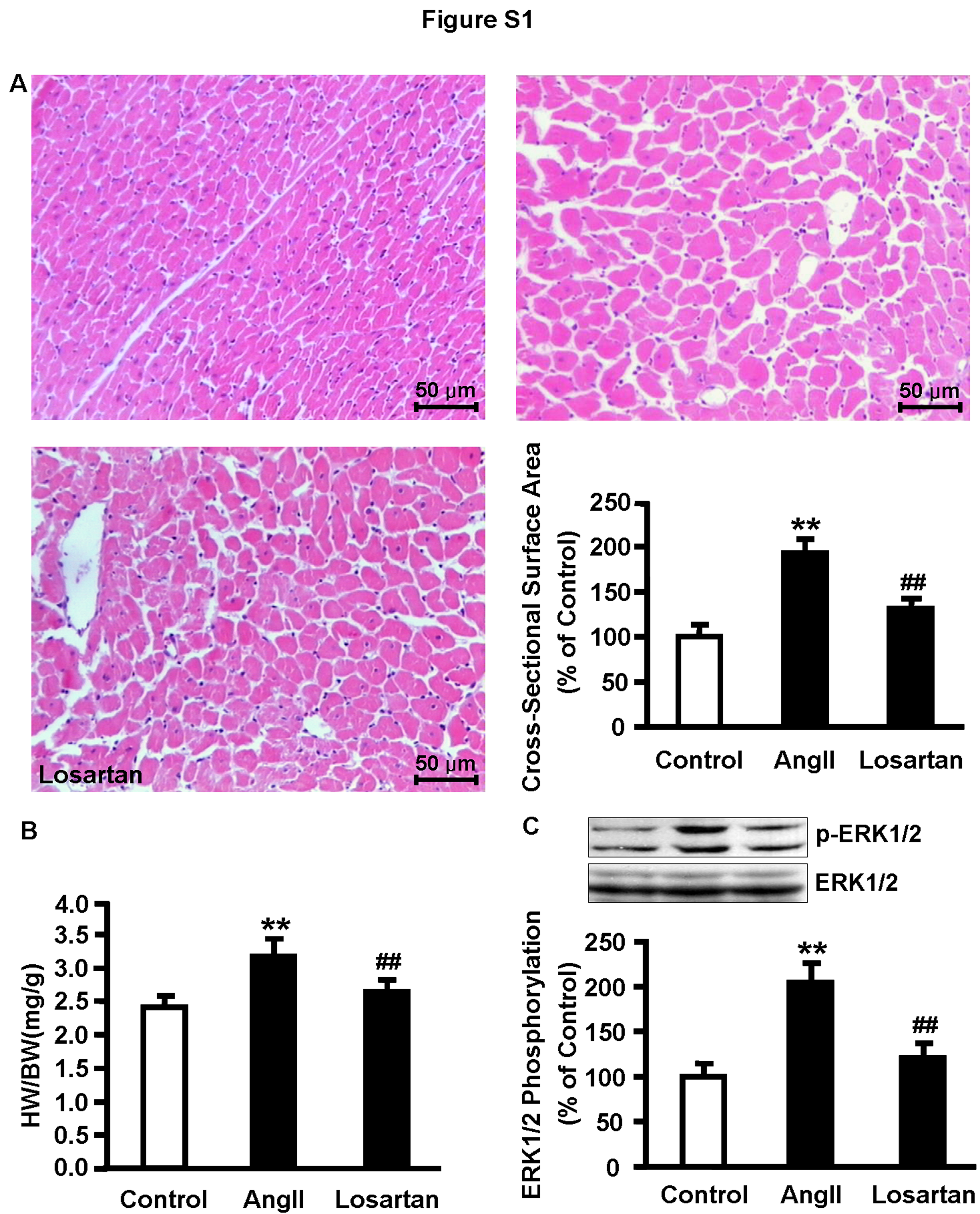

Supplement: Figure S1 — AngII infusion induces cardiac hypertrophy and enhances ERK1/2 phosphorylation. (A) Representative HE staining images and histogram of cardiac myocytes cross-sectional surface area in the control, AngII, and losartan groups (100 myocytes in each section were scanned and averaged). (B) Averaged bar graphs of HW/BW in the control, AngII, and losartan groups. (C) Myocardial extracts were immunoblotted with antibody specific for p-ERK1/2. Membranes were stripped and re-probed to normalize the blotted samples with anti-ERK1/2 antibody. Relative densities of p-ERK1/2 were quantified by scanning densitometry and normalized to percentage of control. Data represent mean ± SE. n = 6 in each group. **P<0.01 vs. control. ## P<0.01 vs. AngII. (TIF) [file pone.0049915.s001.tif]

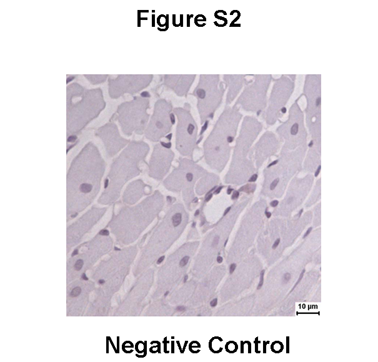

Supplement: Figure S2 — The negative control image of AdipoR1 immunostaining. Deparaffined heart sections (5 µm thickness) were incubated with PBS overnight at 4°C, then horseradish peroxidase (HRP)-conjugated secondary antibody. Immunoreactions were visualized with 3-3′ diaminobenzidine tetrahydrochloride. The nuclei were counterstained with hematoxylin. Cell nuclei is shown in blue. Scale bar represents 10 µm. (TIF) [file pone.0049915.s002.tif]

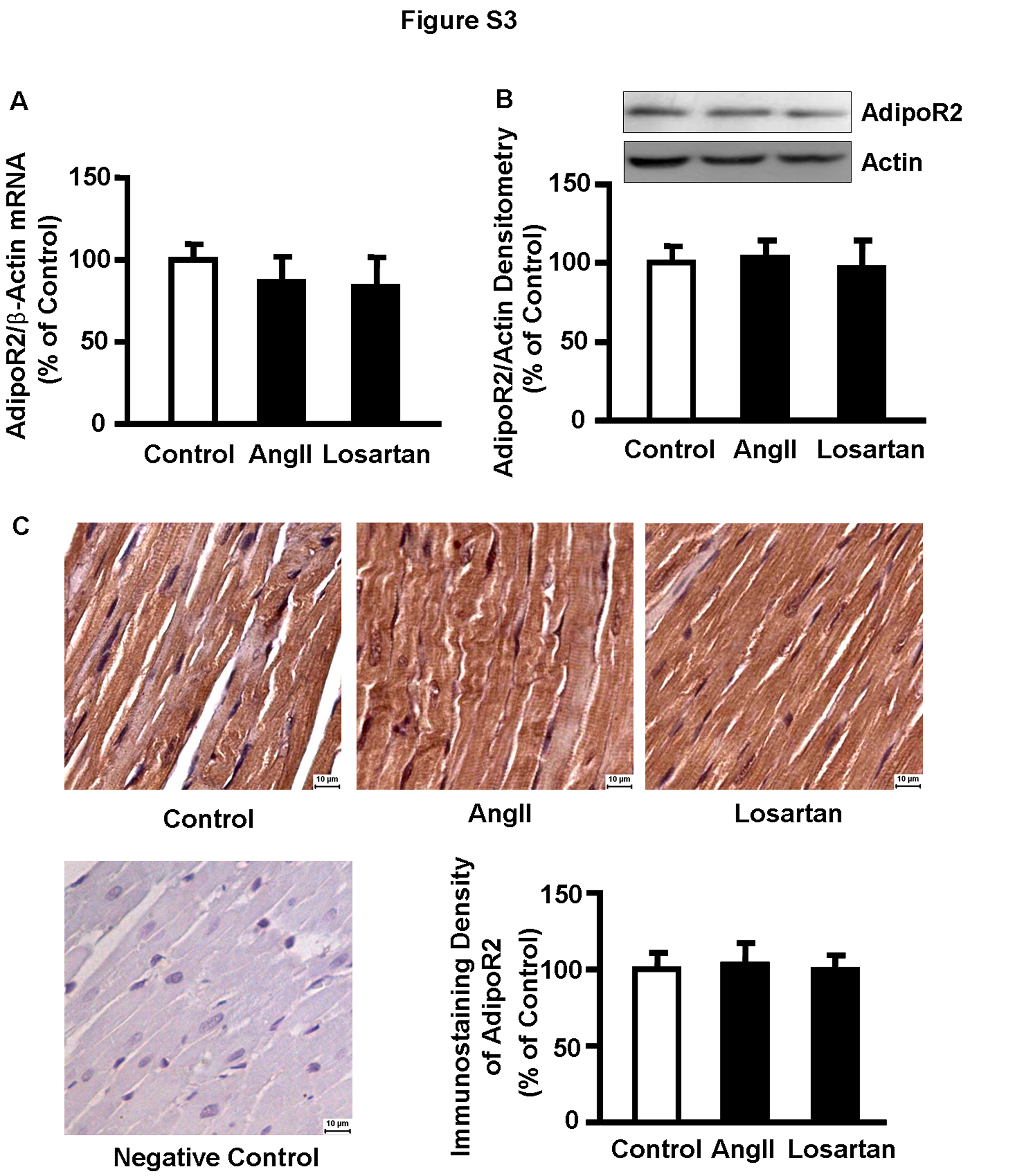

Supplement: Figure S3 — AdipoR2 expression in the control, AngII, and losartan groups. (A) Levels of AdipoR2 mRNA were analyzed by qRT-PCR, and β-actin was used as an internal control. (B) Expression of AdipoR2 protein was determined by Western blot analysis. (C) Representative immunostaining images and averaged bar graphs of AdipoR2 density. AdipoR2 is shown in brown and cell nuclei in blue. Scale bar represents 10 µm (6 fields in each sample were scanned and averaged). Data represent mean ± SE. n = 6 in each group. (TIF) [file pone.0049915.s003.tif]

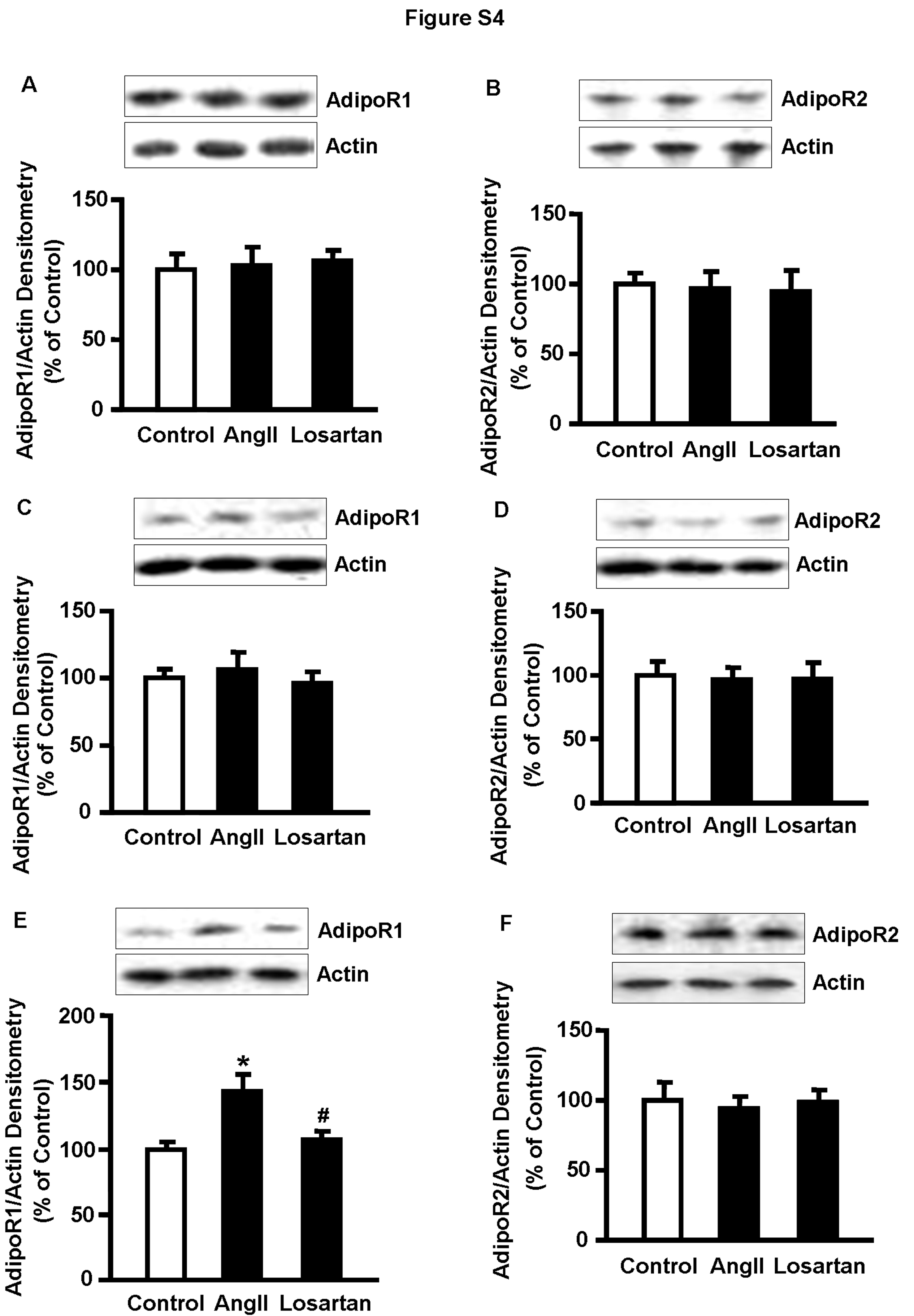

Supplement: Figure S4 — Effect of AngII on AdipoRs expression in skeletal muscle, adipose tissue, and blood vessels. Protein lysates from skeletal muscle (A, B), adipose tissue (C, D), and blood vessels (E, F) were immunoblotted with antibody specific for AdipoR1 (A, C, E) or AdipoR2 (B, D, F). Blots were reprobed with actin to confirm equal loading. Data represent mean ± SE. n = 6 in each group. *P<0.05 vs. control. # P<0.05 vs. AngII. (TIF) [file pone.0049915.s004.tif]

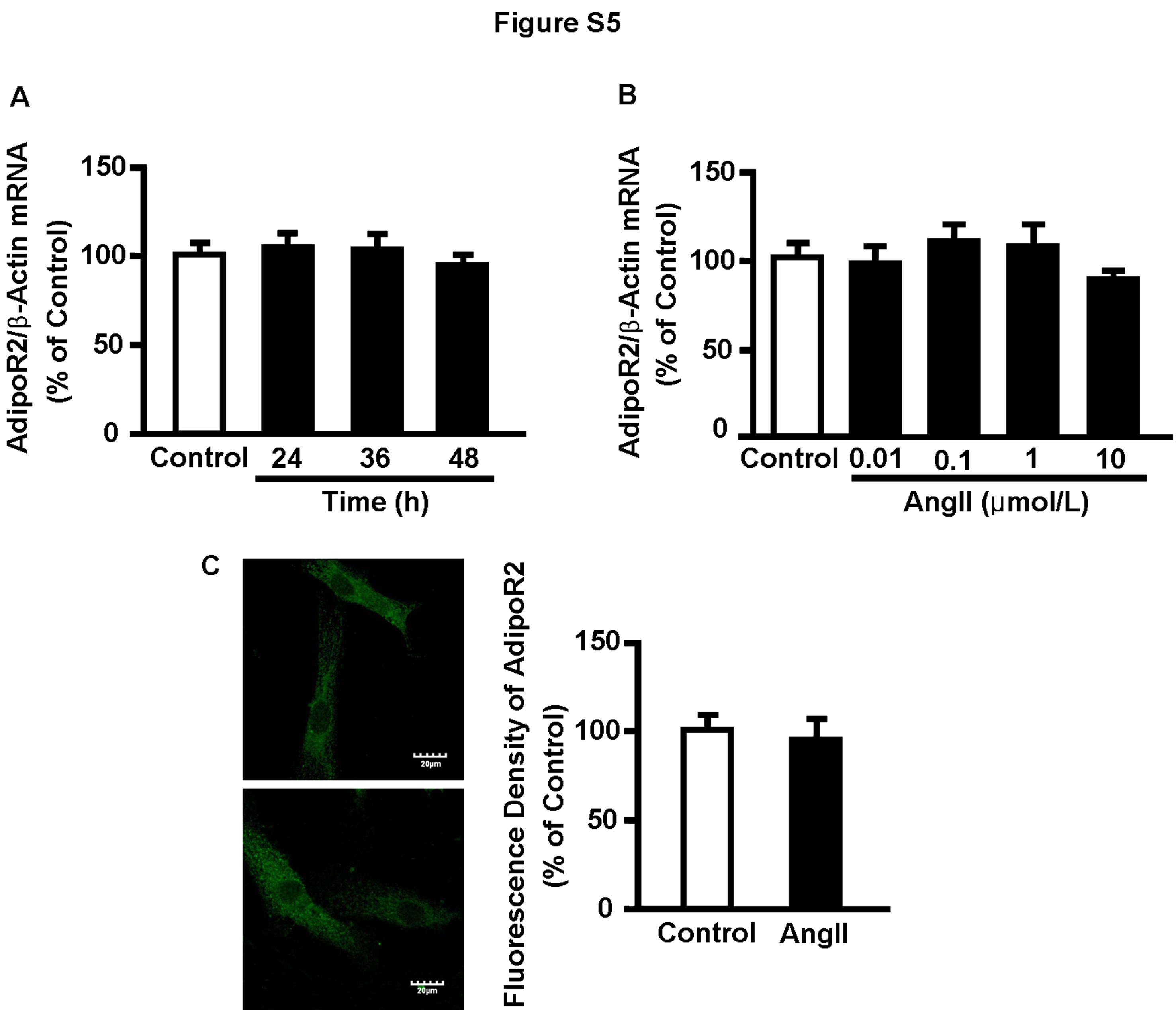

Supplement: Figure S5 — Effect of AngII on AdipoR2 expression in NRVMs. (A, B) NRVMs were incubated with 0.1 µmol/L AngII for the indicated times, or treated with AngII at the indicated concentrations for 48 h. Levels of AdipoR2 mRNA were analyzed by qRT-PCR. (C) Representative immunofluorescence images and averaged bar graphs of AdipoR2 density in unstimulated control NRVMs and cells treated with 0.1 µmol/L AngII for 48 h. Green fluorescence signals represent AdipoR2 protein. Scale bar represents 20 µm (6 fields in each sample were scanned and averaged). Data represent mean ± SE of three independent experiments. (TIF) [file pone.0049915.s005.tif]

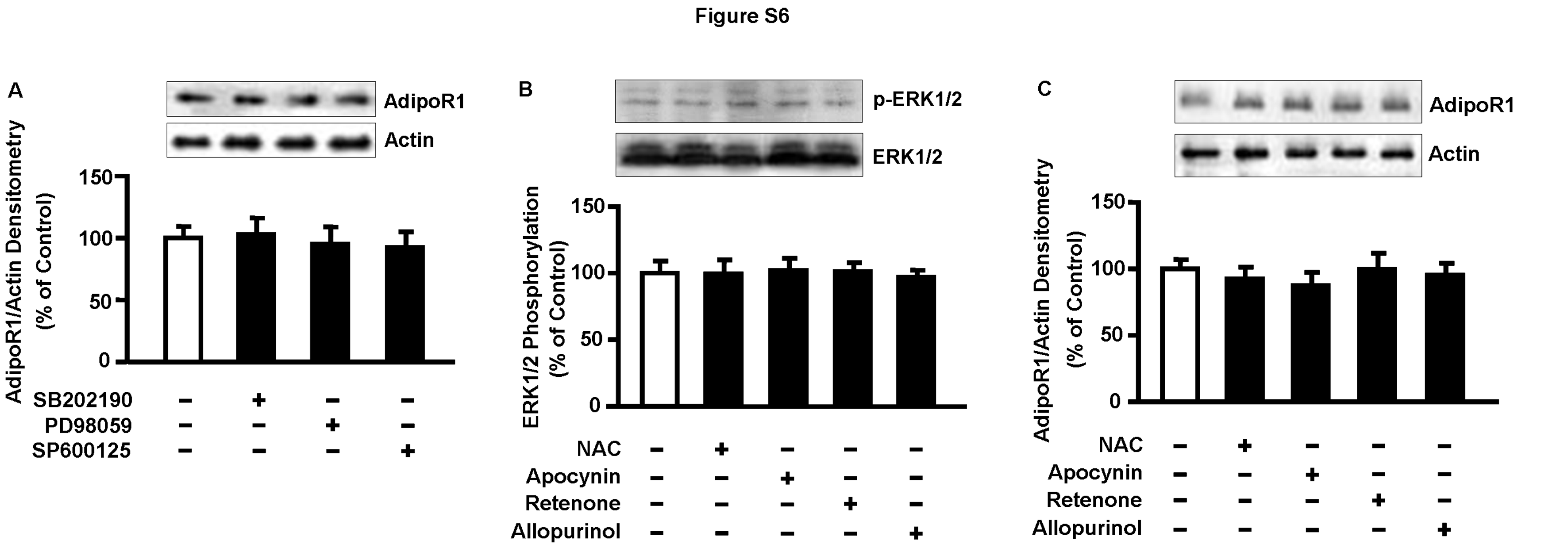

Supplement: Figure S6 — Effect of pharmacological inhibitors of MAPK, ROS scavengers, and the specific inhibitors of ROS-producing enzymes on ERK1/2 phosphorylation or AdipoR1 expression. (A) NRVMs were treated with SB202190 (5 µmol/L), PD98059 (10 µmol/L), or SP600125 (10 µmol/L) for 1 h. Western blot was performed to detect levels of AdipoR1 and actin. (B, C) NRVMs were treated with NAC (10 mmol/L), apocynin (100 µmol/L), rotenone (10 µmol/L), or allopurinol (100 µmol/L) for 1 h. Western blot was performed to detect levels of p-ERK1/2 (B) and AdipoR1 (C). Data represent mean ± SE of three independent experiments. (TIF) [file pone.0049915.s006.tif]

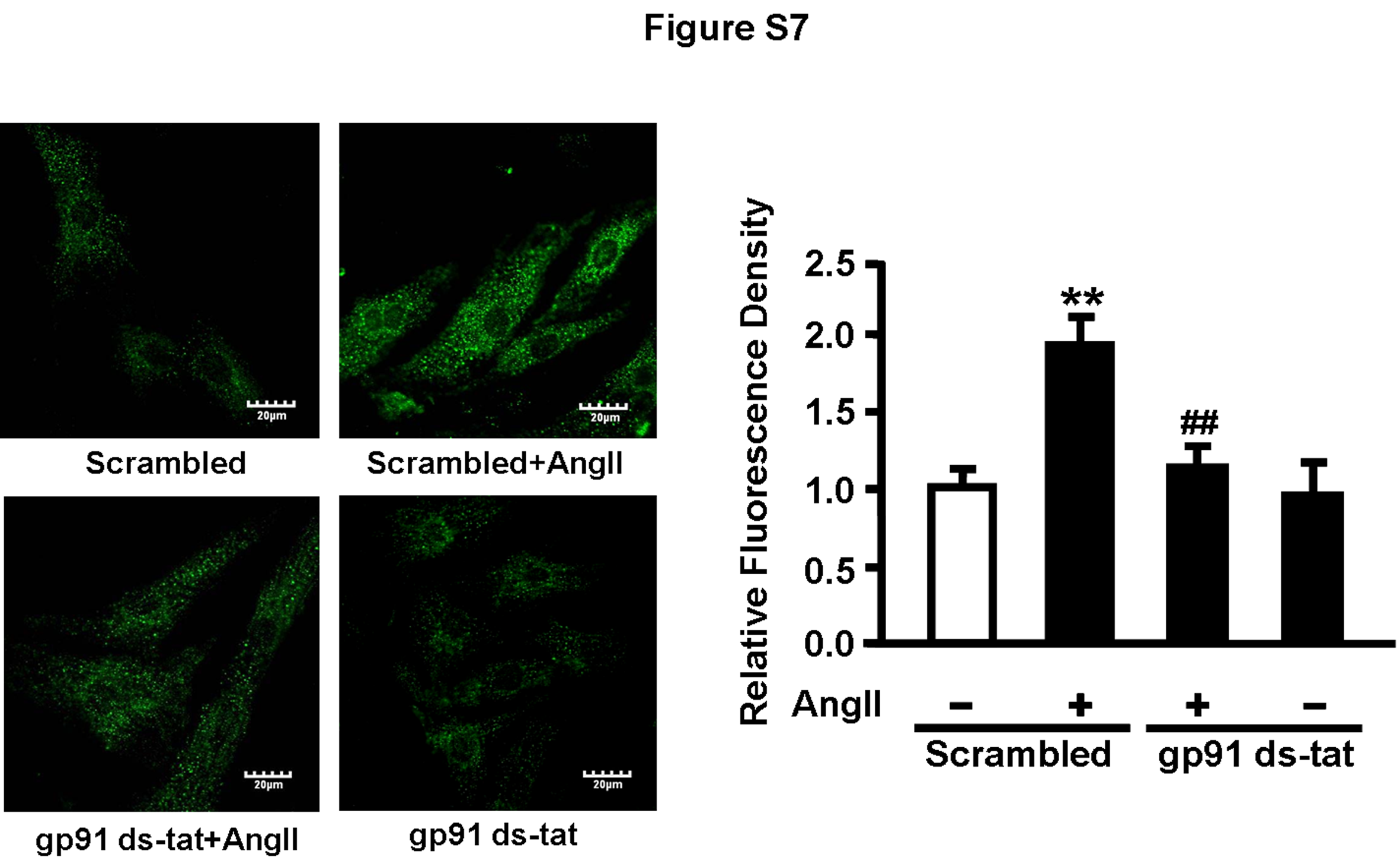

Supplement: Figure S7 — Representative fluorescence images and histogram of relative fluorescence density of ROS. NRVMs were pretreated with 10 µmol/L gp91 ds-tat or scrambled gp91 ds-tat for 1 h, followed by DCF-DA incubation for 30 min, and then stimulated with 0.1 µmol/L AngII for 30 min. DCF fluorescence was visualized using confocal microscopy. The green color represents ROS and the fluorescence density was normalized to cell count. Scale bar represents 20 µm (6 fields in each sample were scanned and averaged). Data represent mean ± SE of three independent experiments. **P<0.01 vs. control. ## P<0.01 vs. AngII. (TIF) [file pone.0049915.s007.tif]
