# Supplementary material for: Angiotensin II Reduces Cardiac AdipoR1 Expression through AT1 Receptor/ROS/ERK1/2/c-Myc Pathway
Source: PLoS One. 2013 Jan 22;8(1):e49915. doi: 10.1371/journal.pone.0049915 (PMC3551944; doi:10.1371/journal.pone.0049915)
Supplement: Table S1 — Hemodynamic parameters with control, angiotensin II, and losartan treatment. AngII, angiotensin II; SBP, systolic blood pressure; DBP, diastolic blood pressure; dP/dt max, peak rate of left ventricular pressure increase; dP/dt min, peak rate of left ventricular pressure decrease; LVEDP, left ventricular end-diastolic pressure. Data are mean ± SE. **P<0.01 vs. control. ## P<0.01 vs. AngII. (DOC) [file pone.0049915.s008.doc]

Table S1. Hemodynamic parameters with control, angiotensin II, and losartan treatment

|  | Control  (n = 6) | AngII  (n = 6) | Losartan  (n = 6) |
| --- | --- | --- | --- |
| SBP (mmHg) | 111.0 ± 12.8 | 206.3 ± 9.6** | 129.7 ± 8.0^##^ |
| DBP (mmHg) | 73.7 ± 3.8 | 153.2 ± 16.1** | 89.8 ± 5.4^##^ |
| d*P*/d*t* max (mm Hg/s) | 4393.2 ± 128.2 | 8386.5 ± 203.1** | 5154.0 ± 132.6^##^ |
| d*P*/d*t* min (mm Hg/s) | -3475.8 ± 144.2 | -6002.7 ± 212.4** | -4201.0 ± 110.5^##^ |
| LVEDP (mmHg) | 7.2 ± 0.3 | 11.6 ± 0.5** | 8.3 ± 0.4^##^ |

AngII, angiotensin II; SBP, systolic blood pressure; DBP, diastolic blood pressure; d*P*/d*t* max, peak rate of left ventricular pressure increase; d*P*/d*t* min, peak rate of left ventricular pressure decrease; LVEDP, left ventricular end-diastolic pressure. Data are mean ± SE. ***P*<0.01 vs. control. ^##^*P*<0.01 vs. AngII.
